# In-patient Expenditure Between 2012 and 2020 Concerning Patients With Liver Cirrhosis in Chongqing: A Hospital-Based Multicenter Retrospective Study

**DOI:** 10.3389/fpubh.2022.780704

**Published:** 2022-03-08

**Authors:** Juntao Tan, Xuewen Tang, Yuxin He, Xiaomei Xu, Daoping Qiu, Jianfei Chen, Qinghua Zhang, Lingqin Zhang

**Affiliations:** ^1^Medical Records and Statistics Room, People's Hospital of Chongqing Banan District, Chongqing, China; ^2^Department of Cardiology, People's Hospital of Chongqing Banan District, Chongqing, China; ^3^Department of Medical Administration, People's Hospital of Chongqing Banan District, Chongqing, China; ^4^Department of Gastroenterology, The Fifth People's Hospital of Chengdu, Chengdu, China; ^5^Department of Infectious Diseases, The First Affiliated Hospital of Chongqing Medical University, Chongqing, China; ^6^Department of Science and Education, People's Hospital of Chongqing Banan District, Chongqing, China; ^7^Department of Biomedical Equipment, People's Hospital of Chongqing Bishan District, Chongqing, China

**Keywords:** liver cirrhosis, medical expenditure, multiple linear regression, time trends, China

## Abstract

**Background:**

Liver cirrhosis is a major global health and economic challenge, placing a heavy economic burden on patients, families, and society. This study aimed to investigate medical expenditure trends in patients with liver cirrhosis and assess the drivers for such medical expenditure among patients with liver cirrhosis.

**Methods:**

Medical expenditure data concerning patients with liver cirrhosis was collected in six tertiary hospitals in Chongqing, China, from 2012 to 2020. Trends in medical expenses over time and trends according to subgroups were described, and medical expenditure compositions were analyzed. A multiple linear regression model was constructed to evaluate the factors influencing medical expenditure. All expenditure data were reported in Chinese Yuan (CNY), based on the 2020 value, and adjusted using the year-specific health care consumer price index for Chongqing.

**Results:**

Medical expenditure for 7,095 patients was assessed. The average medical expenditure per patient was 16,177 CNY. An upward trend in medical expenditure was observed in almost all patient subgroups. Drug expenses were the largest contributor to medical expenditure in 2020. A multiple linear regression model showed that insurance type, sex, age at diagnosis, marital status, length of stay, smoking status, drinking status, number of complications, autoimmune liver disease, and the age-adjusted Charlson comorbidity index score were significantly related to medical expenditure.

**Conclusion:**

Conservative estimates suggest that the medical expenditure of patients with liver cirrhosis increased significantly from 2012 to 2020. Therefore, it is necessary to formulate targeted measures to reduce the personal burden on patients with liver cirrhosis.

## Introduction

Liver cirrhosis is a form of liver dysfunction resulting from multiple factors. The common causes are infection with hepatitis viruses (such as hepatitis B virus[HBV] and hepatitis C virus[HCV]) and alcohol-related liver disease (1). Liver cirrhosis is the 11th most common cause of death worldwide, with ~2 million people dying of liver cirrhosis every year. It has been reported that >630,000 patients had a diagnosis of liver cirrhosis in the United States, with a prevalence rate of ~0.27% and a cirrhosis-related mortality rate of 26.4% per 2-year interval between 1999 and 2010 (2). The progression of compensated liver cirrhosis can lead to decompensation, and on this basis, it can progress to hepatocellular carcinoma. The 5 year cumulative incidence of cirrhosis ranges 8–20% in untreated chronic hepatitis B virus patients. Among those with cirrhosis, the 5 year cumulative risk of hepatic decompensation is 20% (3). The annual risk of HCC in patients with cirrhosis has been reported to be 2–5% (4). According to a study conducted in 2010, liver cirrhosis has become a major global health and economic challenge (5). The economic burden of patients with liver cirrhosis includes both direct medical expenditure (involving drugs and other hospitalization expenses) and indirect medical expenditure (involving an inability to work or a low work efficiency, and a reduced quality of life) (6). Several studies estimate the burden of chronic liver disease in the United States to be in the range of $2.5 billion, with the indirect cost of $10.6 billion (7, 8). Therefore, there is an urgent need for prevention strategies, and the implementation of effective measures should be based on evidence from health economic analyses.

China has the greatest disease burden of liver cirrhosis in the world (9). In China, the number of patients with liver cirrhosis accounts for 20% of the total number of patients with chronic liver disease, most of whom are aged between 20 and 50 years (10). Approximately 50% of deaths due to liver cancer and 15% of deaths due to liver cirrhosis worldwide occur in China (11). In China, although the prevalence of HBV in the general population had dropped to 7.2% by 2006, there remained 97 million HBV carriers (12). Approximately 150 million people are infected with HCV worldwide, of whom ~ 10 million are Chinese patients. Currently, there is no effective cure. Treatment mainly focuses on etiological and symptomatic management, which can only alleviate the disease (13, 14). It is not possible to predict the development and prognosis of liver cirrhosis and determine whether the received treatment measures are effective; therefore, patients are likely to experience illness uncertainty in relation to the disease (15). Illness uncertainty is likely to lead to the deterioration of patients' medical behavior and to interruption of disease treatment. Furthermore, it can seriously affect the rehabilitation process and disease prognosis, increase the risk of readmission, and ultimately increase the economic burden on patients. According to recent reports, the readmission rate for patients with liver cirrhosis has ranged from 20 to 37% for 30 days hospitalization and from 21 to 53% for 90 days hospitalization (16–19). The high readmission rate seriously affects patient quality of life, leading to increased medical expenditures.

Few studies have examined medical expenditure trends in patients with liver cirrhosis in China. Therefore, this study aimed to analyze data collected from multicenter medical institutions to estimate the medical expenditure related to liver cirrhosis between 2012 and 2020, and assess the drivers for such medical expenditure among patients with liver cirrhosis. The study results may help guide medical decision-making and provide better medical protection for such patients.

## Methods

### Data Source

Study data were obtained from electronic medical records of 6 tertiary hospitals on the Big Data Platform of Medical Data Research Institute of Chongqing Medical University. At present, the platform has collected more than 40 million electronic medical records from seven tertiary hospital of Chongqing, and desensitized all information related to patient privacy. The study obtained data on insurance type, sex, age at diagnosis, marital status, length of stay (LOS), smoking status, drinking status, number of complications, etiology (hepatitis B virus, hepatitis C virus, alcoholic liver disease, and autoimmune liver disease), and the age-adjusted Charlson comorbidity index (ACCI) score. The Ethics Committee of the People's Hospital of Chongqing Banan District approved the study. Written informed consent for participation was not required for this study due to its retrospective design, and the study was undertaken in accordance with national legislation and institutional requirements.

### Inclusion and Exclusion Criteria

Target patients defined as patients initially diagnosed liver cirrhosis. The diagnosis of liver cirrhosis is confirmed by liver biopsy, clinical, biochemical, and imaging data or past medical records, and the diagnosis is in accordance with the “Chinese guidelines on the management of liver cirrhosis” (20).

Inclusion criteria comprised the following: (i) patients aged ≥18 years, and (ii) hospitalizations for liver cirrhosis.

Exclusion criteria comprised the following: (i) patients were excluded if basic personal or medical expenditure information was not available, or clinical information such as insurance type, LOS, and overall medical expenditure was incomplete; (ii) if the LOS was ≤ 1 d; (iii) if the last hospital discharge was not between January 1, 2012, and December 31, 2020. The study selection process is depicted in a flow chart (see Supplementary Figure S1 for details).

### Measures

The main outcome measure of this study was medical expenditure per patient. 'Medical expenditure per patient' defined as the medical expenditure of per clinical visit. The indicator of medical expenditure included medical expenditure per patient and medical expenditure per day. Medical expenditure included examinations and laboratory tests, treatment and surgery, drugs, blood products, along with other expenditure for beds and nursing, oxygen, and heating. The insurance type chosen by individuals reflects the economic burden borne by individuals and families. In this study, insurance type was divided into the following five categories: urban employee medical insurance (UEMI), new cooperative medical scheme (NCMS), urban resident medical insurance (URMI), other medical insurance, and full self-payment.The number of complications was defined as the total number of complications affecting patients out of the following: hepatic encephalopathy, gastrointestinal bleeding, ascites, and bacterial infection. The ACCI score was based on the Charlson comorbidity index, which was developed by Charlson et al. to measure baseline comorbid conditions (21, 22).

### Statistical Analysis

Due to the long time span of this study, when comparing medical expenditure, all expenditure data were reported in Chinese Yuan (CNY) based on the 2020 value, which was adjusted using the year-specific personal health care consumer price index (CPI) of Chongqing. We first performed univariate analyses to determine the significance of observed differences in medical expenditure using a two-sample Student's t-test or an ANOVA test after logarithm transition. We then constructed a multiple linear regression model to examine the factors associated with medical expenses per patient. Furthermore, we also described the time trends of medical expenditure and other important measures, the time trend of medical expenditure according to patient subgroups, and the proportional breakdown of medical expenditure. R software (version 4.0.2, Vienna, Austria) and SPSS 22.0 statistical software were used to conduct analyses. The threshold for statistical significance was set at *P* <0.05 (two-tailed tests).

## Results

### Patient Characteristics

For the 2012–2020 study period, 7,095 patients with liver cirrhosis (males, 69.26%) were included in our study (Table 1). The median age at diagnosis was 57 years (P25-P75: 49-67). Cigarette smokers and alcohol consumers accounted for 36.26 and 38.29% of patients, respectively. Patients spent a median LOS of 9 days (P25-P75: 5-14). In total, 91.81% of the patients were married. Most patients had medical insurance cover (72.48%), of which the largest proportion was URMI (41.21%). We also provide the socio-demographic and clinical-pathological characteristics of the 7,095 selected patients, 2012–2020 (Supplementary Table S1).

**Table 1:** Characteristics of the 7,095 selected patients with liver cirrhosis.

**Variable**	**Number of patients [cases (%)]**
Insurance type	
UEMI	2,112 (29.77)
URMI	2,924 (41.21)
NCMS	106 (1.50)
Other insurance	186 (2.62)
Full self-pay	1,767 (24.90)
Sex	
Female	2,181 (30.74)
Male	4,914 (69.26)
Age at diagnosis (years)	
≤ 49	1,883 (26.54)
50–57	1,695 (23.89)
58–67	1,785 (25.16)
≥68	1,732 (24.41)
Marital status	
Married	6,499 (91.60)
Unmarried	238 (3.35)
Other	358 (5.05)
Length of stay (days)	
≤ 5	1,795 (25.30)
6–9	2,172 (30.61)
10–14	1,531 (21.58)
≥15	1,597 (22.51)
Smoking status	
No	4,522 (63.74)
Yes	2,573 (36.26)
Drinking status	
No	4,378 (61.71)
Yes	2,717 (38.29)
Number of complication	
0	2,691 (37.93)
1	4,262 (60.07)
≥2	142 (2.00)
Hepatitis B virus	
No	3,460 (48.77)
Yes	3,635 (51.23)
Hepatitis C virus	
No	6,845 (96.48)
Yes	250 (3.52)
Alcoholic liver disease	
No	6,622 (93.33)
Yes	473 (6.67)
Autoimmune liver disease	
No	6,906 (97.34)
Yes	189 (2.66)
ACCI score
≤ 4	3,369 (47.48)
5	1,608 (22.66)
6	1,037 (14.62)
≥7	1,081 (15.24)

### Medical Expenditure According to Patient Subgroups

Univariate analysis showed that expenditure differed according to insurance type, sex, age at diagnosis, marital status, LOS, smoking status, drinking status, number of complications, autoimmune liver disease, and the ACCI score (*P* <0.05). As more recent data could better reflect the current and future situation, expenditure data for the final 3 years (2018–2020) was also analyzed (Table 2). The results showed that the average expenditure of most subgroups between 2018 and 2020 was higher than that between 2012 and 2020. Multiple linear regression results showed that, compared with the URMI insurance type, the average UEMI medical expenditure was 3.8% higher between 2012 and 2020. Compared with the number of complications (*n* = 0), the number of complications (*n* = 1) was 9.5% higher between 2012 and 2020, whereas the increase in the number of complications (≥2, 22.7%) was even greater. Table 3 shows the results of multiple linear regression.

**Table 2:** Subgroup analysis of medical expenditure for liver cirrhosis per patient.

**Variable**	**Expenditure per patient during 2012–2020 (CNY)**	**Statistics**	***P***	**Expenditure per patient during 2018–2020 (CNY)**	**Statistics**	***P***
Overall	16,177 (15,796–16,559)			16,848 (16,297–17,398)		
Insurance type		90.432	<0.001^a^		64.836	<0.001^a^
UEMI	18,504 (17,292–18,817)			18,976 (17,916–20,037)		
URMI	17,824 (17,215–18,432)			19,029 (18,087–19,972)		
NCMS	16,024 (12,831–19,217)			23,300 (16,193–30,408)		
Other insurance	12,162 (10,405–13,920)			17,940 (10,527–25,354)		
Full self-pay	11,641 (11,040–12,243)			11,437 (10,698–12,177)		
Sex		−2.327	0.020^b^		−1.591	0.112^b^
Female	15,431 (14,778–16,083)			16,113 (15,158–17,067)		
Male	16,509 (16,041–16,977)			17,173 (16,501–17,846)		
Age at diagnosis (years)		8.053	<0.001^a^		7.498	<0.001^a^
≤ 49	17,598 (16,813–18,383)			18,871 (17,634–20,107)		
50–57	15,797 (15,031–16,563)			16,080 (15,067–17,092)		
58–67	15,263 (14,547–15,978)			15,830 (14,773–16,886)		
≥68	15,948 (15,174–16,722)			16,808 (15,788–17,828)		
Marital status		6.931	0.001^a^		4.062	0.017^a^
Married	16,356 (15,955–16,756)			17,085 (16,497–17,674)		
Unmarried	14,477 (12,393–16,561)			14,397 (11,878–16,915)		
Other	14,068 (12,569–15,567)			15,040 (13,146–16,935)		
Length of stay (days)		1403.984	<0.001^a^		869.477	<0.001^a^
≤ 5	7,427 (7,122–7,732)			7,627 (7,210–8,045)		
6–9	11,986 (11,551–12,422)			12,428 (11,788–13,069)		
10–14	17,219 (16,529–17,909)			17,554 (16,591–18,516)		
≥15	30,715 (29,625–31,805)			34,983 (33,305–36,660)		
Smoking status		−4.428	<0.001^b^		−3.427	0.001^b^
No	15,633 (15,167–16,100)			16,339 (15,667–17,012)		
Yes	17,133 (16,477–17,790)			17,790 (16,834–18,745)		
Drinking status		−3.230	0.001^b^		−2.224	0.026^b^
No	15,905 (15,417–16,393)			16,679 (15,976–17,382)		
Yes	16,616 (16,006–17,227)			17,132 (16,247–18,017)		
Number of complication		5.935	0.003^a^		3.429	0.032^a^
0	15,239 (14,660–15,819)			16,007 (14,296–17,717)		
1	16,686 (16,178–17,194)			17,151 (16,470–17,831)		
≥2	18,694 (15,627–21,761)			19,968 (15,779–23,597)		
Hepatitis B virus		−1.638	0.101^b^		0.632	0.527^b^
No	15,733 (15,198–16,268)			16,802 (15,982–17,621)		
Yes	16,600 (16,058–17,143)			16,887 (16,144–17,630)		
Hepatitis C virus		−1.103	0.271^b^		−3.058	0.003^b^
No	16,096 (15,711–16,482)			16,660 (16,103–17,216)		
Yes	18,396 (16,002–20,790)			21,307 (18,026–24,588)		
Alcoholic liver disease		−0.662	0.508^b^		−0.657	0.511^b^
No	16,170 (15,776–16,564)			16,878 (16,299–17,458)		
Yes	16,285 (14,774–17,795)			16,531 (14,777–18,285)		
Autoimmune liver disease		−3.081	0.002^b^		−3.456	0.001^b^
No	16,067 (15,683–16,450)			16,685 (16,127–17,243)		
Yes	20,230 (17,339–23,121)			20800 (17,669–23,931)		
ACCI score		3.717	0.011^a^		9.308	<0.001^a^
≤ 4	16,417 (15,860–16,975)			17,080 (16,233–17,926)		
5	15,237 (14,480–15,995)			15,321 (14,225–16,416)		
6	15,798 (14,808–16,788)			16,186 (14,787–17,585)		
≥7	17,192 (16,159–18,225)			18,637 (17,337–19,937)		

*All expenditure data were presented as mean with 95% confidence interval in parentheses; CNY, Chinese Yuan.*

*^a^ANOVA test after logarithm transition;*

*^b^Two-sample Student t test after logarithm transition*.

**Table 3:** Predictors of medical expenditure concerning patients with liver cirrhosis.

**Variable**	**Analysis 1: medical expenditure during** **2012–2020**^**a**^			**Analysis 2: medical expenditure during** **2018–2020**^**a**^		
	**Confficient (S.E.)**	**95%CI**		**Confficient (S.E.)**	**95%CI**	
Insurance type						
UEMI	Reference			Reference		
URMI	0.042 (0.008)	0.026	0.057	0.035 (0.011)	0.014	0.055
NCMS	0.003 (0.027)	−0.050	0.056	0.141 (0.045)	0.054	0.229
Other Insurance	−0.151 (0.021)	−0.192	−0.110	−0.025 (0.063)	−0.149	0.099
Full self-pay	−0.125 (0.009)	−0.143	−0.107	−0.145 (0.012)	−0.169	−0.122
Sex						
Female	Reference			/	/	/
Male	0.005 (0.008)	−0.011	0.022	/	/	/
Age at diagnosis (years)						
≤ 49	Reference			Reference		
50–57	−0.029 (0.009)	−0.047	−0.011	−0.040 (0.012)	−0.064	−0.015
58–67	−0.069 (0.013)	−0.095	−0.043	−0.071 (0.017)	−0.105	−0.037
≥68	−0.074 (0.015)	−0.104	−0.044	−0.083 (0.019)	−0.121	−0.046
Marital status						
Married	Reference			Reference		
Unmarried	−0.037 (0.018)	−0.073	−0.001	−0.049 (0.022)	−0.092	−0.006
Other	−0.040 (0.015)	−0.070	−0.010	−0.040 (0.018)	−0.076	−0.003
Length of stay (days)						
≤ 5	Reference			Reference		
6–9	0.208 (0.009)	0.191	0.225	0.215 (0.011)	0.193	0.237
10–14	0.365 (0.009)	0.346	0.383	0.372 (0.012)	0.348	0.397
≥15	0.611 (0.009)	0.593	0.629	0.655 (0.013)	0.630	0.680
Smoking status						
No	Reference			Reference		
Yes	0.012 (0.009)	−0.006	0.031	0.011 (0.012)	−0.013	0.035
Drinking status						
No	Reference			Reference		
Yes	0.010 (0.009)	−0.008	0.028	0.005 (0.012)	−0.018	0.029
Number of complication						
0	Reference			Reference		
1	0.027 (0.007)	0.013	0.041	−0.004 (0.010)	−0.023	0.015
≥2	0.052 (0.024)	0.006	0.099	0.002 (0.031)	−0.059	0.064
Hepatitis C virus						
No	/	/	/	Reference		
Yes	/	/	/	0.077 (0.022)	0.034	0.121
Autoimmune liver disease						
No	Reference			Reference		
Yes	0.055 (0.021)	0.014	0.096	0.071 (0.023)	0.026	0.116
ACCI score						
≤ 4	Reference			Reference		
5	0.026 (0.013)	0.001	0.051	0.020 (0.016)	−0.013	0.052
6	0.037 (0.015)	0.008	0.066	0.035 (0.018)	−0.001	0.071
≥7	0.055 (0.014)	0.026	0.083	0.059 (0.018)	0.024	0.093
Adjusted R^2^ %	0.424	/	/	0.451	/	/

*^a^The logarithmic transformation of the medical expenditure data was performed in the multivariate regression analysis*.

### Time Trends of Medical Expenditure and Other Important Measures (2012–2020)

**Figure 1:**
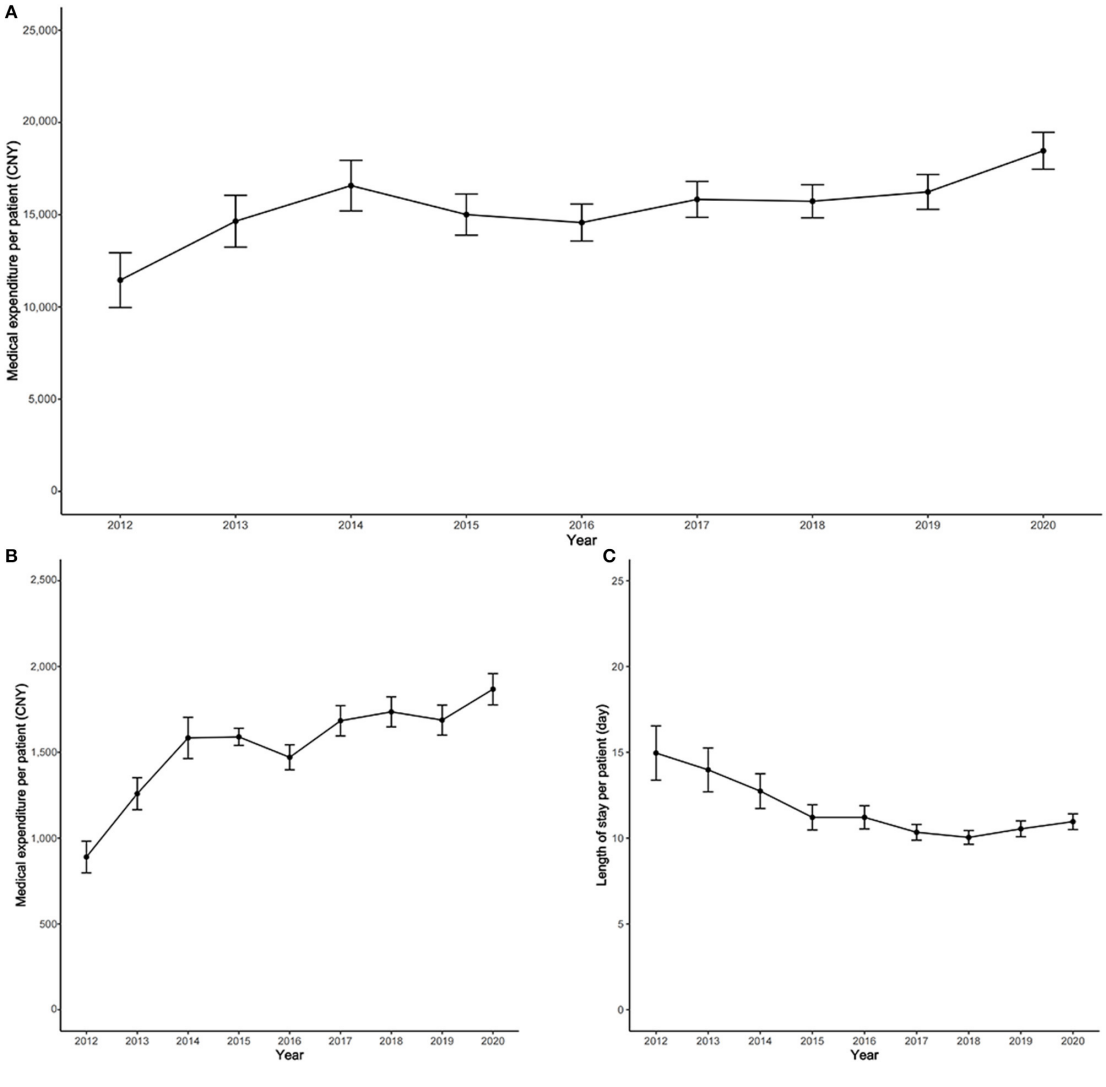
Time trend of medical expenditure and related factors for cirrhosis, 2012–2020. **(A)** Time trend of overall average medical expenditure per patient; **(B)** time trend of daily average medical expenditure; **(C)** time trend of the length of stay per patient.

Figure 1 shows medical expenditure time trends and related factors for patients with cirrhosis in the 2012–2020 period. The overall average expenditure per patient increased 7.65% per year from 11,453 CNY in 2012 to 18,461 CNY in 2020 (Figure 1A). The average daily expenditure significantly increased a further 13.74% per year from 890 CNY in 2012 to 1,868 CNY in 2020 (Figure 1B). The year 2018 was a turning point in the LOS per patient, with the LOS per patient decreasing 5.48% per year before 2018, and then gradually increasing after 2018 (Figure 1C). At the same time, we also provide the medical expenditure of included patients by different study center (Supplementary Table S2).

### Medical Expenditure Time Trends According to Patient Subgroups (2012–2020)

**Figure 2:**
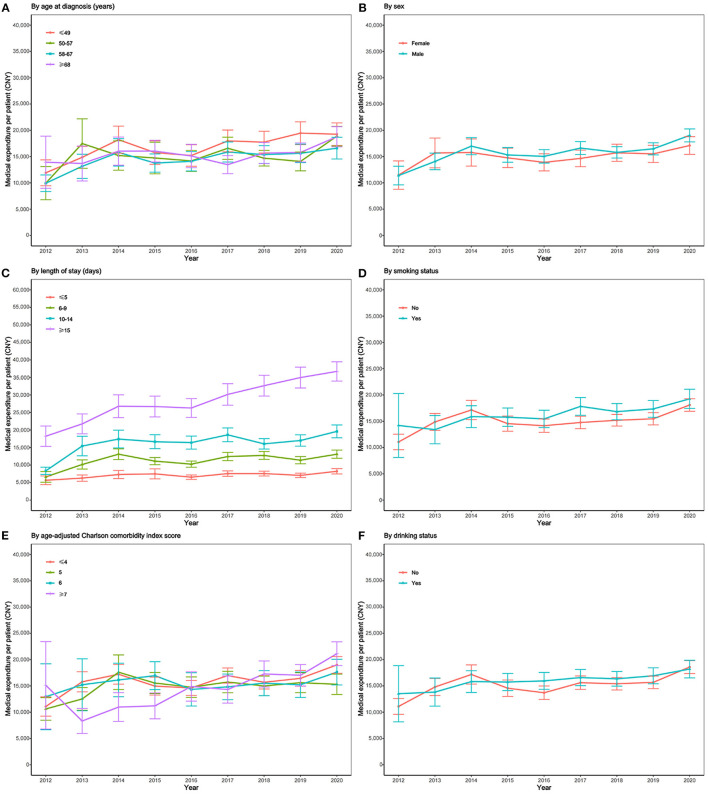
Subgroup analysis on time trend of medical expenditure for cirrhosis per patient, 2012–2020. **(A)** Time trend of medical expenditure stratified by age at diagnosis; **(B)** time trend of medical expenditure stratified by sex; **(C)** time trend of medical expenditure stratified by LOS; **(D)** time trend of medical expenditure stratified by smoking status; **(E)** time trend of medical expenditure stratified by ACCI score; **(F)** time trend of medical expenditure stratified by drinking status.

The average medical expenditure time trend differed according to age at diagnosis, sex, LOS, smoking status, the ACCI score, and alcohol consumption status (Figure 2). The average medical expenditure per patient aged <49 years at diagnosis was higher than that for patients aged 50–57 years, 58–67 years, and ≥68 years. The average medical expenditure per patient for those aged 50–57 years, 58–67 years, and ≥68 years did not differ significantly (Figure 2A). The average medical expenditure per male patient was higher than that for females, and the average medical expenditure per male patient in 2020 was 1.11 times that per female patient (Figure 2B). The average medical expenditure per patient for LOS ≥15 days was considerably higher than that for LOS ≤ 5 days, 6–9 days, and 10–14 days, and the gap between LOS ≥15 days and other LOS subgroups gradually increased (Figure 2C). Further details are also presented according to smoking status, the ACCI score, and alcohol consumption status (Figures 2D–F).

### The Proportional Breakdown of Medical Expenditure (2012–2020)

**Figure 3:**
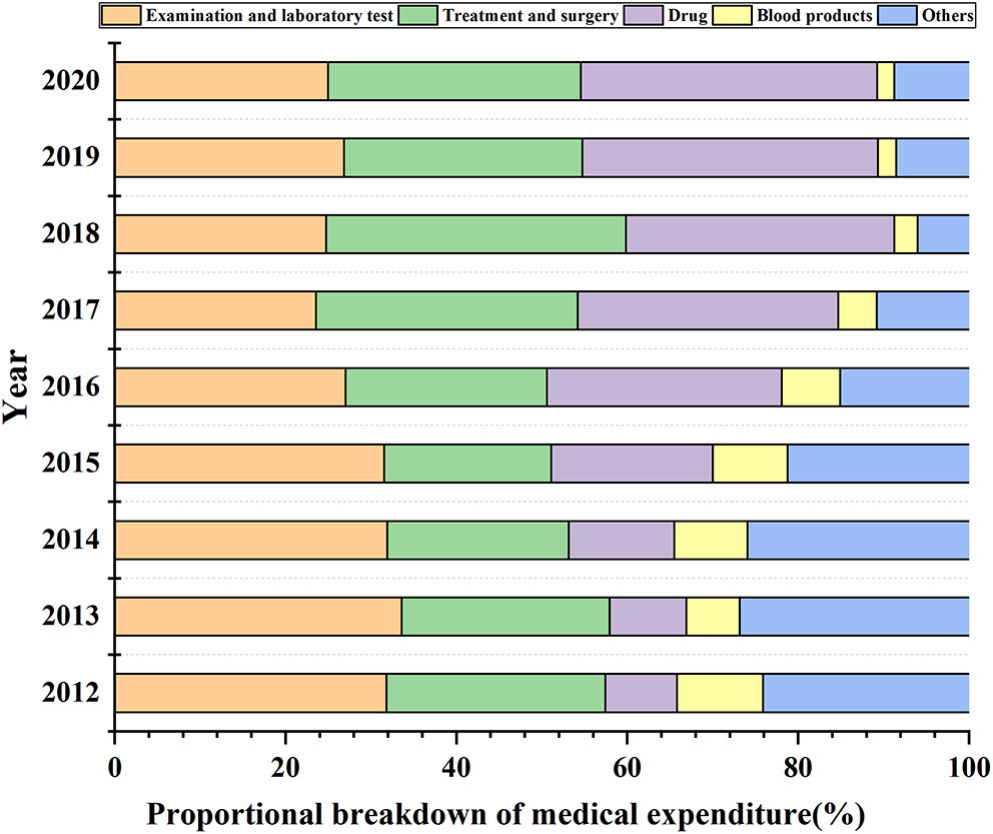
The proportional breakdown of medical expenditures for cirrhosis per patient. Others include the expenditures for bed and nursing, oxygen, heating, and so on.

The proportion of drug expenses gradually increased during 2012-2020. In particular, it increased from 8% in 2012 to 35% in 2020 (Figure 3). Conversely, the proportion of blood product expenses gradually decreased during 2012-2020. In particular, it decreased from 10% in 2012 to 2% in 2020. Expenses for examinations and laboratory tests, treatment, and surgery were maintained at >20% during these 9 years.

## Discussion

In this study, the medical expenditure of patients with liver cirrhosis gradually increased from 2012 to 2020, and the average annual growth rate in total medical expenditure was 7.65%. This increase in medical expenditure implied that some indirect expenses, such as patients' non-medical expenses, patient family care, lost work, transportation, and board and lodging were also likely to have increased. Our results showed that liver cirrhosis imposed a heavy economic burden on both individuals and society and that medical expenditure varied greatly among different subgroups of patients with liver cirrhosis.

In our study, the average medical expenditure for liver cirrhosis in 2020 was 18,461 CNY. According to data from the National Bureau of Statistics in 2019 (data not updated for 2020), Chongqing's gross domestic product (GDP) ranked 17th among the 31 provinces in China, which was at a medium level (23). Therefore, our results may be closer to the average national cost. According to the average household population (2.6 persons) and the per capita disposable income of rural residents (16,021 CNY) in 2019, the average medical cost per patient with cirrhosis was 44.3% of the average annual family income of rural households, indicating that the economic burden imposed due to liver cirrhosis was relatively high for individual patients' families in China, especially for low-income families. In addition, once advanced liver cirrhosis develops, a patient's medical expenditure will be higher. A cost study showed that compared with the mild stage of liver cirrhosis, costs increased 1.6 times with decompensated cirrhosis, 1.9 times with hepatocellular carcinoma, and 3.4 times with liver transplant, respectively (24). Xu et al. reported that the average medical expenditure of patients with liver cancer from 2009 to 2011 was as high as 35,248 CNY (95% confidence interval, 34,304–36,193) (25).

We found that the medical expenditure of patients with UEMI, NCMS, and URMI insurance types was significantly higher than that of those with other medical insurance and full self-pay. The UEMI is the most established medical insurance program in China, dating back to the mid-1990s (26). At the beginning of the 21st century, the Chinese government established two more insurance programs, namely, the NCMS for rural residents and the URMI for self-employed and unemployed urban residents (27). It has been reported that the medical expenses of older adults had increased from an average of $204.77 in 2005 to $696.23 in 2014. By 2014, the proportion of medical expense reimbursement for older adults in the rural NCMS had increased significantly from 30.6% in 2005 to 56.1% in 2014 (28). In 2008, the number of individuals insured with URMI was 118.26 million, which had increased to 376.89 million in 2015. The per capita pooling fund of URMI was 140 CNY in 2008, decreasing to 130 CNY in 2009, and then increasing annually, reaching 515 CNY in 2015 (29). In view of the increasing demand for higher quality medical care, the NCMS and the URMI still have great potential for improvement. With an aging population and the gradual increase in medical expenses and other complex factors, the UEMI, NCMS, and URMI are facing pressure on fund payments. Therefore, it is necessary to formulate specific policies to improve the social medical insurance system so that more people can access affordable medical care services.

We found that the average medical expenditure per patient increased 1.61 times from 2012 to 2020, which was consistent with results reported in other studies. Desai et al. analyzed the national data of inpatients with liver cirrhosis from 2008 to 2014 and found that hospitalization expenses increased 30.2% from 2008 to 2014, reaching USD $7.37 billion (30). We also found that the LOS time trend per patient began to increase gradually after 2018. A possible reason for this is that, with improvement in national medical insurance policies and enhanced disease awareness among individuals, an increasing number of patients with severe liver disease (see Supplementary Figure S2A) choose to go to hospital, with a view to improving their quality of life and delaying progression of the disease through treatment (31, 32).

In our study, we compared medical expenditure time trends concerning patients with liver cirrhosis among various patient subgroups. Patients aged ≤ 49 years had the highest average medical expenditure. The population of patients with severe liver diseases such as liver cirrhosis, liver failure, and liver cancer is gradually becoming younger; therefore, these patients usually need more comprehensive treatment measures after the first diagnosis (33, 34). The average medical expenditure was highest when the LOS was ≥15 days. Extending the LOS will inevitably increase the medical expenditure of patients, which is consistent with the results of other studies (35, 36). In addition, we also found that the difference in medical expenditure between a LOS ≥15 days and other LOS subgroups became increasingly larger (see Supplementary Figure S2B). This might be related to more modern medical equipment and more sophisticated medical services. The average medical expenditure of male patients was higher than that of female patients, higher in patients who smoked than in those who did not, and higher in patients who consumed alcohol than in those who did not. This might be attributed to men generally having unhealthy lifestyle habits such as smoking, excess alcohol consumption, and an unhealthy diet (37), all of which seriously affect the metabolic capacity of the liver and increase the risk of aggravation. Therefore, measures need to be taken in response to these challenges, such as increasing the frequency of monitoring for men, encouraging smoking and alcohol consumption cessation, and undertaking active treatment and preventive measures to avoid disease deterioration, thereby reducing the economic burden.

Our data showed that the proportion of drug costs had increased annually, possibly because patients with liver cirrhosis have many complications and are prone to recurring attacks. Patients develop complications such as abdominal infection, upper gastrointestinal bleeding, and hypoproteinemia, and symptomatic treatments such as blood transfusion and albumin transfusion are required (38, 39). Patients with liver cirrhosis have multiple recurrent infections and require treatment with many potent antibiotics. Furthermore, due to the advanced nature of treatment, the frequency of expensive drug use, such as biological drugs, has increased gradually (40), which has led to an increase in the proportion of drug expenses.

This study had several limitations. First, the patient data included in this study were all obtained from six tertiary hospitals in Chongqing, China, which may have led to selection bias and reduced the generality of the data. Second, this study only collected data on hospitalization medical expenditure within the study hospitals; however, some patients may have been diagnosed and treated in other hospitals, meanwhile, we were unable to collect patients' outpatient expenditure information. so our data may underestimate the economic burden of liver cirrhosis. Third, we were also unable to collect patients' income information, so we were unable to analyze the relative financial burden of medical expenditure for patients with liver cirrhosis from 2012 to 2020. Further research is needed to address these limitations and provide a more comprehensive understanding of the medical expenditure of patients with liver cirrhosis.

Nevertheless, a strength of our study was its use of patient-level data, which covered a spectrum of patients and settings across various developmental and socioeconomic levels. Through analyzing the source of medical expenditure, we determined factors that had an important effect on medical expenditure, which can provide a reference for formulating effective measures to reduce the economic burden on individuals, families, society, and the government.

## Conclusion

In conclusion, there is a heavy economic burden on patients with liver cirrhosis, on their families, and on society. Our findings indicated that the medical expenditure of patients with liver cirrhosis increased significantly from 2012 to 2020; therefore, formulating targeted measures to reduce the personal burden on these patients is needed.

## Data Availability Statement

The raw data supporting the conclusions of this article will be made available by the authors, without undue reservation.

## Ethics Statement

The studies involving human participants were reviewed and approved by the Ethics Committee of the People's Hospital of Chongqing Banan District. Written informed consent for participation was not required for this study in accordance with the national legislation and the institutional requirements.

## Author Contributions

JT and XT: concept and design. JT: drafting of the manuscript. JT and YH: collected data. JT and XX: statistical analysis. DQ, QZ, JC, and LZ: administrative, technical, and material support. All authors contributed to the article and approved the submitted version.

## Funding

This study was funded by Chongqing Science and Health Joint Medical Research Project (2020GDRC016).

## Conflict of Interest

The authors declare that the research was conducted in the absence of any commercial or financial relationships that could be construed as a potential conflict of interest.

## Publisher's Note

All claims expressed in this article are solely those of the authors and do not necessarily represent those of their affiliated organizations, or those of the publisher, the editors and the reviewers. Any product that may be evaluated in this article, or claim that may be made by its manufacturer, is not guaranteed or endorsed by the publisher.

## Abbreviations

HBV: hepatitis B virus
HCV: hepatitis C virus
LOS: length of stay
ACCI: age-adjusted Charlson comorbidity index
UEMI: urban employee medical insurance
NCMS: new cooperative medical scheme
URMI: urban resident medical insurance
CNY: Chinese Yuan
CPI: consumer price index
GDP: gross domestic product.
