# Supplementary material for: In-patient Expenditure Between 2012 and 2020 Concerning Patients With Liver Cirrhosis in Chongqing: A Hospital-Based Multicenter Retrospective Study
Source: Front Public Health. 2022 Mar 8;10:780704. doi: 10.3389/fpubh.2022.780704 (PMC8957842; doi:10.3389/fpubh.2022.780704)
Supplement: Supplementary file 1 [file Data_Sheet_1.docx]

**Supplementary information**

**Figure S1** Flow of inclusions and exclusions


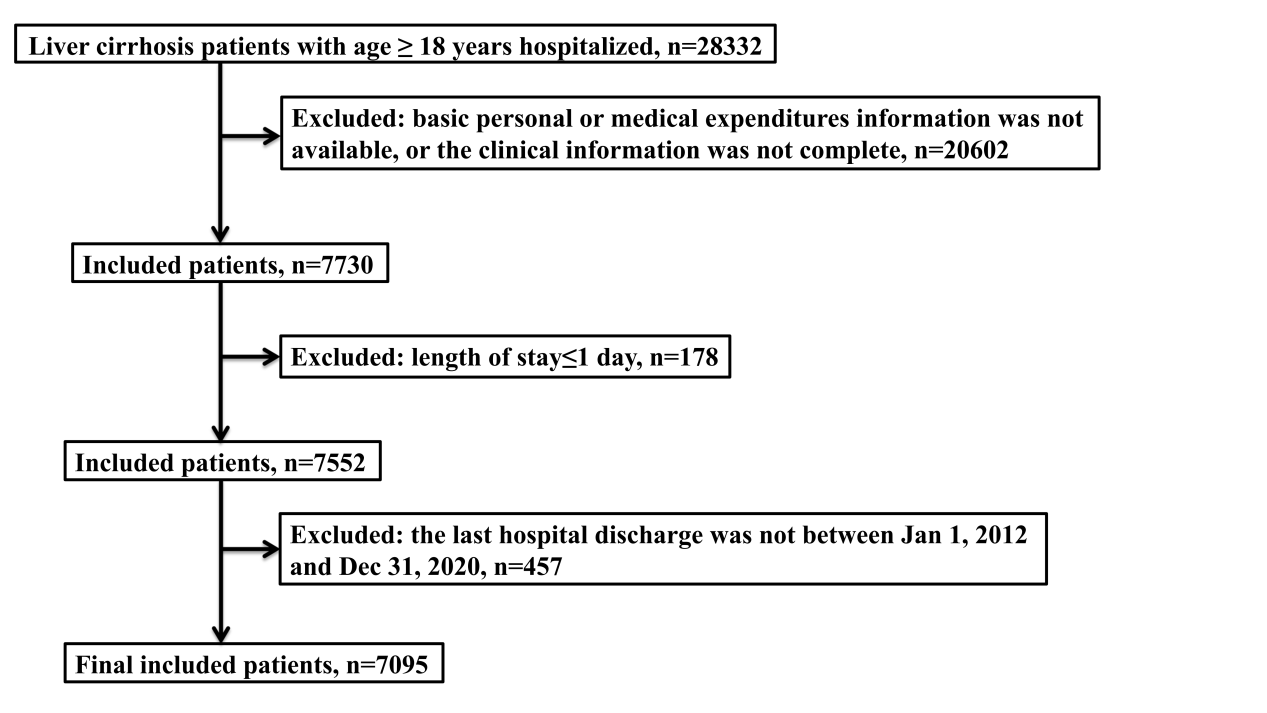


**Table S1** Socio-demographic and clinical-pathological characteristics of the 7095 selected patients, 2012-2020

| Variable | Number of patients | | | | | | | | | |
| --- | --- | --- | --- | --- | --- | --- | --- | --- | --- | --- |
|  | Total | 2012 | 2013 | 2014 | 2015 | 2016 | 2017 | 2018 | 2019 | 2020 |
| Insurance type |  |  |  |  |  |  |  |  |  |  |
| UEMI | 2112 | 5 | 86 | 145 | 176 | 185 | 268 | 379 | 411 | 457 |
| URMI | 2924 | 22 | 158 | 227 | 265 | 283 | 399 | 489 | 549 | 532 |
| NCMS | 106 | 41 | / | / | 4 | 7 | 15 | 11 | 3 | 25 |
| Other insurance | 186 | 86 | 15 | 28 | 20 | 9 | 9 | 8 | 5 | 6 |
| Full self-pay | 1767 | 12 | 17 | 20 | 37 | 185 | 322 | 395 | 380 | 399 |
| Sex |  |  |  |  |  |  |  |  |  |  |
| Female | 2181 | 55 | 88 | 114 | 145 | 234 | 302 | 400 | 425 | 418 |
| Male | 4914 | 111 | 188 | 306 | 357 | 435 | 711 | 882 | 923 | 1001 |
| Age at diagnosis (years) |  |  |  |  |  |  |  |  |  |  |
| ≤49 | 1883 | 60 | 110 | 157 | 170 | 180 | 274 | 278 | 306 | 348 |
| 50-57 | 1695 | 28 | 42 | 65 | 88 | 162 | 241 | 349 | 319 | 401 |
| 58-67 | 1785 | 46 | 72 | 111 | 123 | 175 | 254 | 345 | 359 | 300 |
| ≥68 | 1732 | 32 | 52 | 87 | 121 | 152 | 244 | 310 | 364 | 370 |
| Marital status |  |  |  |  |  |  |  |  |  |  |
| Married | 6499 | 164 | 273 | 388 | 470 | 630 | 943 | 1186 | 1196 | 1249 |
| Unmarried | 238 | 2 | 2 | 8 | 11 | 25 | 23 | 36 | 57 | 74 |
| Other | 358 | / | 1 | 24 | 21 | 14 | 47 | 60 | 95 | 96 |
| Length of stay (days) |  |  |  |  |  |  |  |  |  |  |
| ≤5 | 1795 | 28 | 53 | 97 | 114 | 154 | 257 | 340 | 361 | 391 |
| 6-9 | 2172 | 33 | 67 | 111 | 163 | 221 | 339 | 429 | 395 | 414 |
| 10-14 | 1531 | 40 | 60 | 90 | 106 | 122 | 216 | 275 | 331 | 291 |
| ≥15 | 1597 | 65 | 96 | 122 | 119 | 172 | 201 | 238 | 261 | 323 |
| Smoking status |  |  |  |  |  |  |  |  |  |  |
| No | 4522 | 147 | 223 | 260 | 253 | 415 | 594 | 823 | 842 | 965 |
| Yes | 2573 | 19 | 53 | 160 | 249 | 254 | 419 | 459 | 506 | 454 |
| Drinking status |  |  |  |  |  |  |  |  |  |  |
| No | 4378 | 144 | 218 | 260 | 249 | 389 | 577 | 780 | 814 | 947 |
| Yes | 2717 | 22 | 58 | 160 | 253 | 280 | 436 | 502 | 534 | 472 |
| Number of complication |  |  |  |  |  |  |  |  |  |  |
| 0 | 2691 | 155 | 240 | 325 | 305 | 190 | 211 | 433 | 437 | 395 |
| 1 | 4262 | 11 | 36 | 95 | 196 | 458 | 769 | 838 | 889 | 970 |
| ≥2 | 142 | / | / | / | 1 | 21 | 33 | 11 | 22 | 54 |
| Hepatitis B virus |  |  |  |  |  |  |  |  |  |  |
| No | 3460 | 165 | 250 | 340 | 320 | 235 | 293 | 604 | 618 | 635 |
| Yes | 3635 | 1 | 26 | 80 | 182 | 434 | 720 | 678 | 730 | 784 |
| Hepatitis C virus |  |  |  |  |  |  |  |  |  |  |
| No | 6845 | 166 | 272 | 416 | 496 | 641 | 969 | 1234 | 1306 | 1345 |
| Yes | 250 | / | 4 | 4 | 6 | 28 | 44 | 48 | 42 | 74 |
| Alcoholic liver disease |  |  |  |  |  |  |  |  |  |  |
| No | 6622 | 157 | 270 | 412 | 497 | 641 | 952 | 1196 | 1232 | 1265 |
| Yes | 473 | 9 | 6 | 8 | 5 | 28 | 61 | 86 | 116 | 154 |
| Autoimmune liver disease |  |  |  |  |  |  |  |  |  |  |
| No | 6906 | 165 | 276 | 417 | 497 | 659 | 1003 | 1234 | 1303 | 1352 |
| Yes | 189 | 1 | / | 3 | 5 | 10 | 10 | 48 | 45 | 67 |
| ACCI score |  |  |  |  |  |  |  |  |  |  |
| ≤4 | 3369 | 95 | 166 | 241 | 267 | 347 | 504 | 594 | 542 | 613 |
| 5 | 1608 | 41 | 63 | 89 | 116 | 156 | 230 | 286 | 336 | 291 |
| 6 | 1037 | 21 | 33 | 57 | 83 | 85 | 149 | 187 | 200 | 222 |
| ≥7 | 1081 | 9 | 14 | 33 | 36 | 81 | 130 | 215 | 270 | 293 |

**Table S2** The description of medical expenditure of included patients by different study center

| Hospital | Number of patients [cases (%)] | Expenditure per patient |
| --- | --- | --- |
| A | 2,264(31.91) | 17,584(16,961-18,206) |
| B | 1,967(27.72) | 21,871(20,939-22,804) |
| C | 1,320(18.61) | 9,130(8,674-9,586) |
| D | 650(9.16) | 15,099(13,962-16,237) |
| E | 623(8.78) | 9,344(8,655-10,033) |
| F | 271(3.82) | 15,726(13,885-17,568) |
| Total | 7,095(100.00) | 16,177(15,796-16,559) |

**Figure S2** Time trend of number of patients with severe liver diseases and those of difference in medical expenditure


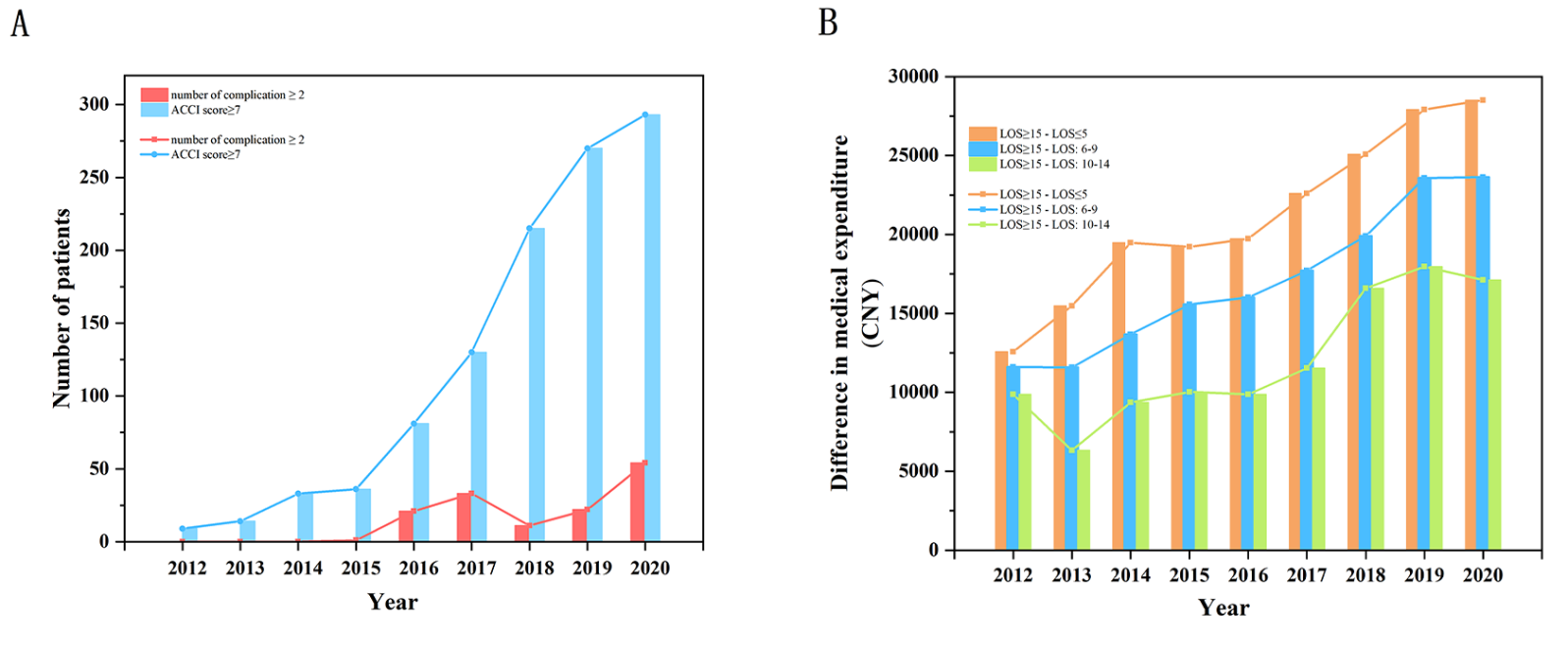


**A** Time trend of number of patients with severe liver diseases; **B** time trend of difference in medical expenditure
